# Performance of federated learning-based models in the Dutch TAVI population was comparable to central strategies and outperformed local strategies

**DOI:** 10.3389/fcvm.2024.1399138

**Published:** 2024-07-05

**Authors:** Tsvetan R. Yordanov, Anita C. J. Ravelli, Saba Amiri, Marije Vis, Saskia Houterman, Sebastian R. Van der Voort, Ameen Abu-Hanna

**Affiliations:** ^1^ Department of Medical Informatics, Amsterdam University Medical Centers, University of Amsterdam, Amsterdam, Netherlands; ^2^ Amsterdam Public Health Research Institute, Amsterdam University Medical Centers, University of Amsterdam, Amsterdam, Netherlands; ^3^ Informatics Institute, University of Amsterdam, Amsterdam, Netherlands; ^4^ Department of Cardiology, Amsterdam University Medical Centers, University of Amsterdam, Amsterdam, Netherlands; ^5^ Amsterdam Cardiovascular Sciences Institute, Amsterdam University Medical Centers, University of Amsterdam, Amsterdam, Netherlands; ^6^ Netherlands Heart Registration, Utrecht, Netherlands

**Keywords:** federated learning, multicenter, prediction models, TAVI, distributed machine learning, privacy-preserving algorithms, risk prediction, EHR

## Abstract

**Background:**

Federated learning (FL) is a technique for learning prediction models without sharing records between hospitals. Compared to centralized training approaches, the adoption of FL could negatively impact model performance.

**Aim:**

This study aimed to evaluate four types of multicenter model development strategies for predicting 30-day mortality for patients undergoing transcatheter aortic valve implantation (TAVI): (1) *central*, learning one model from a centralized dataset of all hospitals; (2) *local*, learning one model per hospital; (3) *federated averaging* (*FedAvg*), averaging of local model coefficients; and (4) *ensemble*, aggregating local model predictions.

**Methods:**

Data from all 16 Dutch TAVI hospitals from 2013 to 2021 in the Netherlands Heart Registration (NHR) were used. All approaches were internally validated. For the *central* and federated approaches, external geographic validation was also performed. Predictive performance in terms of discrimination [the area under the ROC curve (AUC-ROC, hereafter referred to as AUC)] and calibration (intercept and slope, and calibration graph) was measured.

**Results:**

The dataset comprised 16,661 TAVI records with a 30-day mortality rate of 3.4%. In internal validation the AUCs of *central*, *local*, *FedAvg*, and *ensemble* models were 0.68, 0.65, 0.67, and 0.67, respectively. The *central* and *local* models were miscalibrated by slope, while the *FedAvg* and *ensemble* models were miscalibrated by intercept. During external geographic validation, *central*, *FedAvg*, and *ensemble* all achieved a mean AUC of 0.68. Miscalibration was observed for the *central*, *FedAvg*, and *ensemble* models in 44%, 44%, and 38% of the hospitals, respectively.

**Conclusion:**

Compared to centralized training approaches, FL techniques such as *FedAvg* and *ensemble* demonstrated comparable AUC and calibration. The use of FL techniques should be considered a viable option for clinical prediction model development.

## 1 Introduction

The increasing adoption of electronic health records (EHRs) across healthcare facilities has led to a wealth of data that can be harnessed for developing prediction models for various medical applications. Such models may improve patient stratification, inform clinical decision-making, and ultimately enhance patient outcomes. In the field of cardiovascular medicine, combining records from multiple centers has successfully been used in training clinical prediction models (CPMs) (1). Such multicenter models tend to generalize better and are more robust than those derived from individual centers. Although models trained on data from a single center may perform well within their local hospital settings, they require a large number of records for training, and their performance often deteriorates when applied to new centers or other patient populations. However, sharing patient data between centers is not always straightforward. Concerns about patient privacy, the implementation of new regulations such as the General Data Protection Regulation (GDPR), and the challenges of integrating data from different centers all pose significant challenges. There is a growing need to implement strategies for training prediction models on multiple datasets without sharing records between them.

Federated learning (FL) has emerged as a promising approach to address this challenge. FL is a machine learning approach that enables multiple parties to build a shared prediction model without needing to exchange patient data.

However, implementing FL comes with its own set of challenges. Aside from logistical and communication issues, an important question is whether FL has a detrimental impact on the quality of learned models (2). While promising, the impact of FL on model quality has yet to be thoroughly examined in various areas of medicine.

Understanding the potential benefits and limitations of FL in developing multicenter prediction models helps facilitate a more effective and privacy-preserving use of electronic patient data in risk prediction. To that end, our analysis investigates the potential of FL as a viable strategy for multicenter prediction model development.

FL has rarely been studied in the cardiovascular context (3–5) and not yet in the transcatheter aortic valve implantation (TAVI) population, which is the focus of this study. TAVI is a relatively new and minimally invasive treatment for severe aortic valve stenosis. The Netherlands Heart Registration (NHR) is a centralized registry that holds records of all cardiac interventions performed in the Netherlands, including those of TAVI patients who are treated in the 16 hospitals performing this operation. Across these 16 hospitals, the TAVI patient population could vary for a number of reasons, such as regional population demographic differences.

Risk prediction models for TAVI patients have been developed using data originating from a single hospital (6) or combining records from multiple centers (1, 7, 8). In a previous study, we evaluated the performance of one such centralized, multicenter TAVI early-mortality CPM and observed the model to have a moderate degree of external performance variability, most of which could be attributed to differences in hospital case-mix (9). However, the performance of such models in an FL approach, compared to a centralized or local approach, remains unknown.

We aimed to evaluate the impact of two important FL techniques: federated averaging (*FedAvg*) (10) and mean ensemble (henceforth referred to as *ensemble*) (11), explained further in the Materials and methods section, on the predictive performance of TAVI risk prediction models. This performance is compared to a *centralized model* and *local* center-specific models (Table 1).

**Table 1:** Method overview of model development strategies with respect to types of data sharing and validation performance evaluation (the main differences and similarities between the four model strategies used in the current experiments are shown).

				Federated learning			
	Model strategy	*Central*	*Local*	*FedAvg*		*Ensemble*	
Aspect				No recalibration	Recalibration	No recalibration	Recalibration
Sharing	Predictor data	Yes (by design)	No	No	No	No	No
	Outcome data	Yes (by design)	No	No	Yes	No	Yes
	Model parameters	Yes (by design)	No	Yes	Yes	No	No
	Predictions	Yes (by design)	No	No	Yes	Yes	Yes
	Optional: other model parameters	Central imputation	No	Local imputation	Local imputation; central recalibration	Local imputation	Local imputation; central recalibration
	Calibration	Yes (by design)	Yes (by design, per center)	No	No	No	No
	Recalibration	No	No	No	Local, central, federated	No	Local, central, federated
Validation	Stacking predictions CV	Per (test) fold	Per (test) fold per center^a^	Per (test) fold	Per (test) fold	Per (test) fold	Per (test) fold
	Stacking predictions LCOA	Per external center	Not Applicable	Per external center	Per external center	Per external center	Per external center
	Obtaining performance	On stacked predictions	On stacked predictions	On stacked predictions	On stacked predictions	On stacked predictions	On stacked predictions

Clarifications—Sharing of predictor data: the predictor variable values of records from a center dataset. Sharing of outcome data: the outcome variable value of records from a center dataset. Sharing of model parameters: the weights and intercepts (coefficients) from a center-learned model. Sharing of predictions: the predicted probabilities from a center-learned model. Sharing of other model parameters (optional): imputation model for missing values, recalibration model. Calibration: does the model fitting process also calibrate the model predictions? Recalibration: recalibration (of any kind) applied to the model after its fitting? Stacking predictions CV: during CV, how were the model predictions from the test folds stacked (combined) before computing performance metrics? Stacking predictions LCOA: during LCOA, how were the model predictions from the test centers stacked (combined) before computing performance metrics? Obtaining performance: when computing performance metrics for a model, what set of predictions were used?

^a^ In the case of local models, for each individual center, the model predictions from all of its test folds during CV were stacked together. Each set of these stacked predictions was then used to obtain the per-center local model performance measures. Pooled performance across all center local models was then calculated with a REMA pooling of the individual center performance results.

## 2 Materials and methods

This study adhered to the Transparent Reporting of a Multivariable Prediction Model for Individual Prognosis or Diagnosis (TRIPOD) statement (12). This study meets all five of the CODE-EHR minimum framework standards for the use of structured healthcare data in clinical research (13).

### 2.1 Dataset

In this nationwide retrospective multicenter cohort study, we included all patients who had a TAVI intervention in any Dutch hospital for the 9-year period from 1 January 2013 to 31 December 2021. Data were collected by the NHR (14). Permission was granted for this study to use the data and include a pseudonymized code indicating the center in the dataset (Supplementary Appendix A).

The outcome of interest was the 30-day post-operative mortality. Mortality data were obtained by checking the regional municipal administration registration, Basisregistratie Personen (BRP).

Patients lacking the outcome measurement of 30-day mortality were excluded.

No ethical approval was needed according to the Dutch central committee of Human Research, as the study only used previously collected cohort registry data. All data in this study were fully anonymized before we accessed them. Approval for this study was granted by the Committee of Research and Ethics of the Netherlands Heart Registry on 2 February 2021.

### 2.2 Model strategies

Four different model development strategies (henceforth referred to as models) were considered in our experiments (Table 1). A *central* model was derived using the combined records from all hospitals (Supplementary Figure S1). The derivation of such a model consists of two steps: (1) performing variable selection from the list of candidate predictor variables (explained further in Section 2.3); and (2) fitting predictor variable coefficients. Leveraging the entire dataset enables capturing relationships between predictors and outcomes across multiple centers. Due to the nature of the central design, all data between hospitals are shared, including individual patient record variables.

In the next strategy, multiple *local* models were trained, one for each hospital's dataset (Supplementary Figure S2). The derivation of each center local model would follow in much the same steps as in the centralized model strategy. As the *local* models are specific to each hospital, they avoid the need to share any data between centers.

In addition to these baselines, we considered two popular FL techniques: *FedAvg* and an *ensemble* model. To conceptualize the idea behind *FedAvg*, one can think of averaging the knowledge of a classroom where students train on their schoolwork and then share their key learnings with a central teacher who combines them to create a better understanding for everyone. In the case of the current study, each participating center trains a local model for one epoch (that is, one pass on all the data) and shares its model parameters with a central server (10). Once each center has shared model parameters, model updates are aggregated by the central server (Supplementary Figure S3). This new aggregated model is then sent back to the centers for further local training in the next epoch. This process continues until convergence or a pre-specified number of epochs is reached.

The *ensemble* model approach is similar to combining votes from a diverse group, where the final prediction is the most popular choice (similar to how a majority vote wins an election). For the *ensemble* model in this case, a local model is fitted on each center's data. The ensemble's prediction for each patient is then formed by averaging the predictions of each local model from all hospitals (Supplementary Figure S4) (15). With this strategy, only hospital-level models are transmitted between the centers.

For all model development strategies, we fitted logistic regression models with Least Absolute Shrinkage and Selection Operator (LASSO) penalization. This approach results in automatically selecting variables deemed predictive of the outcome.

#### 2.2.1 Model recalibration

Table 1 provides a framework for summarizing, among others, the aspect of model recalibration. Ensuring a model is well-calibrated before its application in practice is critical. If a decision is to be made based on a predicted probability from a model, then the predicted probability should be as close as possible to the true patient risk probability. This is what calibration performance measures.

The recalibration aspect describes the addition of a final step to the model derivation process, where recalibration of the intercept and slope of the linear predictor is performed using the model's predictions on the training data. The derived recalibration function is used thereafter whenever the model makes predictions. Specifically, in recalibration, we align the true outcomes from the different centers with their corresponding model predictions followed by fitting the recalibration function. This is done by fitting a logistic regression model in which the predicted 30-day mortality probability is the sole covariate to predict the true 30-day mortality outcome. As listed in Table 1, recalibration could be done in a local, central, or federated manner. In the local case, a recalibration function would be learned for each individual hospital and then used for adjusting model predictions for patients belonging to the corresponding hospital. In the central case, a single recalibration function would be learned on the combined training dataset predictions from all hospitals. In the federated recalibration approach, a federated learning strategy (such as *FedAvg*) would be used to derive a single recalibration function while also avoiding the need to share the uncalibrated predictions between centers.

In our main analysis, we focused on the results from the FL techniques with central recalibration and did not investigate all options, such as learning the recalibration function in a federated manner.

### 2.3 Candidate predictor variables

The TAVI dataset included variables for patient characteristics (e.g., age, sex, and body mass index), lab test results (e.g., serum creatinine), relevant medical history (e.g., chronic lung disease), and procedure characteristics (e.g., access route and use of anesthesia) (Supplementary Table S1). All 33 candidate predictors were collected prior to the intervention. Threshold values for the body surface area (BSA) were used in summarizing patient characteristics.

In all model strategies, we used LASSO to perform automatic variable selection. In the case of *FedAvg*, LASSO was first used on each hospital dataset. Later, the selected predictors from each hospital-local LASSO were aggregated via center-weighted voting and a center agreement strength hyperparameter (Supplementary Methods S1).

### 2.4 Experimental evaluation

We adopted two primary evaluation strategies to fit the type of evaluation: a 10-fold cross-validation (CV) approach for the internal validation and leave-center-out analysis (LCOA) for the geographic validation.

In some cases, a hospital-local model could not be fitted due to the insufficient number of records for the prevalence of outcomes. We compared the performance of the *local* model to the other models only in cases where a *local* model was successfully derived and reported the cases where fitting a *local* model failed.

#### 2.4.1 Cross-validation

For cross-validation, we first randomly partitioned the entire dataset into ten equal subsets, stratified by the outcome. In each iteration of the CV, we utilized nine subsets (90% of the records) for model training and held out the remaining subset (10% of the records) for testing. This process was repeated 10 times, each with a different test set. In the case of *local*, *FedAvg,* and *ensemble*, the entire dataset was first partitioned by hospital, and thereafter each hospital dataset was randomly partitioned into 10 equal subsets stratified by the outcome.

#### 2.4.2 Leave-center-out analysis

For the federated strategies, we conducted a LCOA for a more robust external geographic validation (9). In this approach, we created as many train/test dataset pairs as there were hospitals in the dataset. For each pair, the training set encompassed records from all hospitals but one (the excluded hospital), while the test set solely contained records from the excluded hospital. This method allows us to evaluate how well each model performed when applied to a new center.

#### 2.4.3 Pooling results

In the context of CV, mean metric values and confidence intervals (CIs) for a model were derived from the individual metric results per test set. During this process, the predictive performance of each model was computed separately for each test set, generating 10 metric values. These 10 values were then averaged to arrive at the mean pooled metric, and their standard deviation was used to compute a 95% CI.

During the LCOA, performance was calculated per external hospital and then pooled via random effects meta-analysis (REMA) with hospital as the random effect to give a mean estimate and 95% CI.

#### 2.4.4 Performance metrics

Discrimination was evaluated using the area under the ROC curve (AUC-ROC, henceforth referred to as AUC). The AUC metric summarizes a model's ability to discriminate between events and cases. It involves sensitivity (also called recall in information retrieval) and specificity across all possible threshold values. Calibration was evaluated by the Cox method using the calibration intercept and slope and their corresponding 95% CIs (16). A model's predictions were deemed to be miscalibrated if either (1) the 95% CI for its Cox calibration intercept did not contain the value zero (miscalibration by intercept) or (2) the 95% CI of its intercept did contain the value zero, but the 95% CI for its Cox calibration slope did not contain the value one (miscalibration by the slope) (16).

In addition, calibration graphs showing a model's predicted probabilities vs. the observed frequencies of positive outcomes were drawn for visual inspection.

Net reclassification improvement (NRI) was calculated between the predictions of any two models in either validation strategy (CV and LCOA) (Supplementary Methods S2).

#### 2.4.5 Significance testing

Bootstrapping with 3,000 samples was used to test for a difference in (paired) AUCs between two prediction models (17). This test was run per AUC of each test fold dataset during CV. Analogously, the test was applied per AUC of each external center dataset in the LCOA.

#### 2.4.6 Sensitivity analyses

Apart from the main experimental setup, we considered two additional modifications to it in the form of sensitivity analyses.

First, to see what effect the recalibration step was having on the two FL approaches (*FedAvg* and *ensemble*), we evaluated their performance without recalibration in a sensitivity analysis. In a second sensitivity analysis, we excluded hospitals with a low TAVI volume from the dataset and re-evaluated the models’ performance results. In this case, we defined a low TAVI volume to be any hospital that performed fewer than 10 TAVI procedures in any year of operation after its first year.

### 2.5 Hyperparameter optimization

Hyperparameters for LASSO and *FedAvg* were optimized empirically on the training data (Supplementary Methods S3). For LASSO, we optimized the regularization parameter lambda, while for *FedAvg*, we optimized on the learning rate, number of training epochs, and variable selection agreement strength (Supplementary Methods S1).

### 2.6 Handling of missing data

Variables with more than 30% missing values were not included as predictors.

The remaining missing values were assumed to be missing at random and were imputed using the Chain Equations (18). As shown in Table 1, imputation was done on the center-combined dataset for the *central* model, while for the other models, imputation was handled separately per-center dataset. In both validation strategies (CV and LCOA), missing values were imputed separately on the training and test sets (further information is provided in Supplementary Methods S4).

### 2.7 Fitting final models

From each of the four strategies, a “final” version of their model was fitted using records from the complete dataset. We used the resulting final models to report on and compare the predictor variables selected by each model strategy. In the case of *central* and *FedAvg*, the “final” model comprised of just one single logistic regression model, while for *local*, the “final” model was a set of *h* hospital-local models (where *h* is the number of hospitals in the dataset). The *ensemble* produced a “final” model comprised of *h* local models and one top-level model, which averaged the predictions from the *h* hospital-level models.

### 2.8 Software

All statistical analyses were performed in the R programming language (version 4.2.1) and R studio (version 2023.03.1). The metamean function from the meta package was used for conducting the REMA, and the roc.test function from the pROC package was used for the bootstrap testing for the difference in AUCs between two models. The “mice” package in R was used for imputing missing values (mice version 3.14.0). All the source code used in this analysis was documented and made openly available on GitHub (https://github.com/tsryo/evalFL). Experiments were carried out on a desktop machine with 16 GB of memory and an i7-10700 2.9 GHz processor and took approximately 2 days to run.

## 3 Results

**Figure 1:**
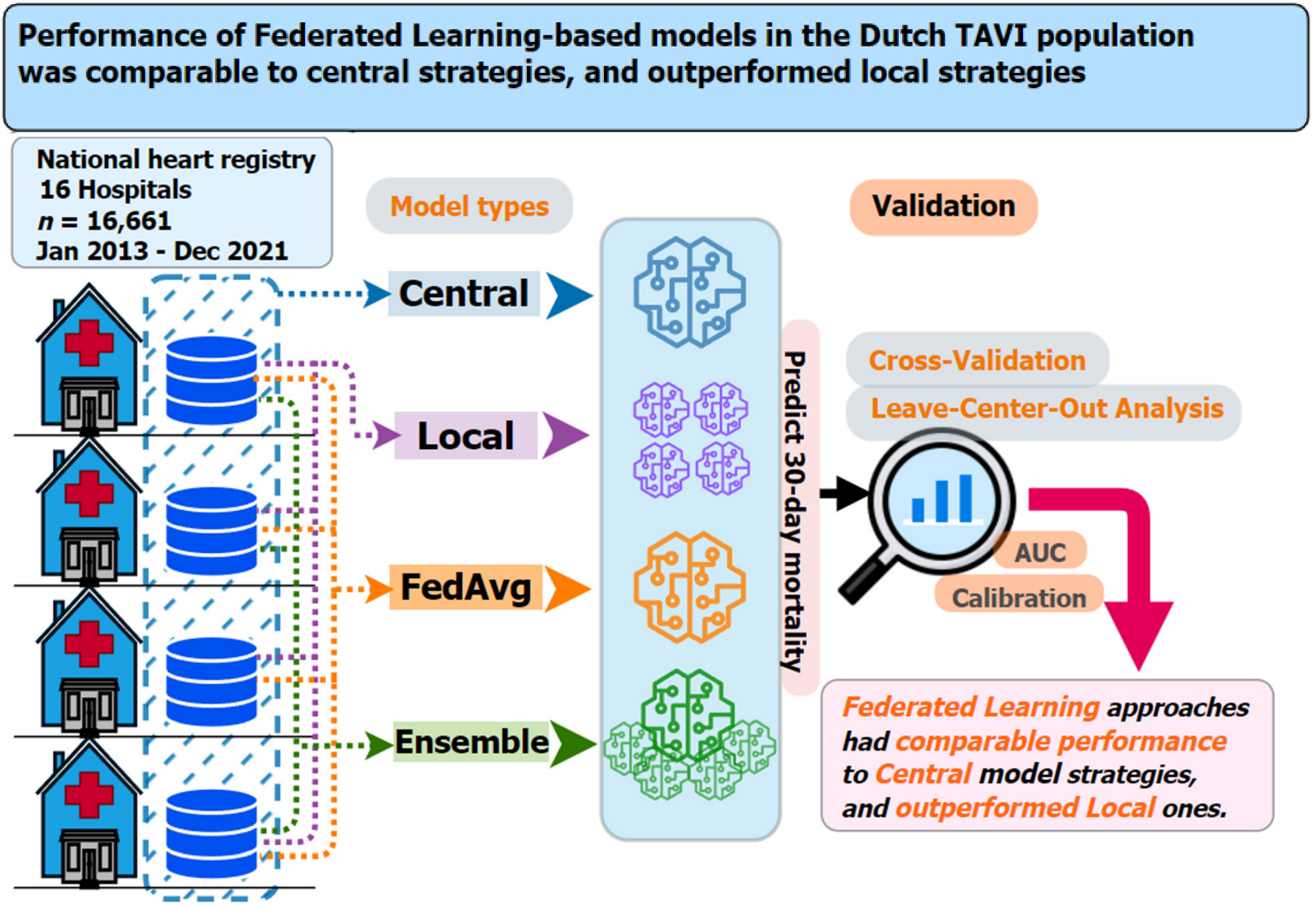
Graphical summary of the dataset used, prediction models considered, validation strategies employed, and main findings for the current study on 30-day mortality risk prediction models for TAVI patients. Key questions are as follows: In the context of multicenter TAVI risk prediction models, what is the impact on model performance from adopting two federated learning strategies (*FedAvg* and *ensemble*) compared to *central* and *local*-only model strategies? Key findings are as follows: Both federated learning strategies (*FedAvg* and *ensemble*) had comparable performance, in terms of discrimination and calibration, to that *central* models and outperformed the *local*-only models. Take-home message is as follows: Use of federated learning techniques should be considered a viable option for TAVI patient clinical prediction model development.

The results in this section are structured into four sub-sections. First, we provide summary statistics of the TAVI dataset and its pre-processing. We then report on the models’ predictive performance measures from cross-validation and LOCA. Third, we report on selected predictor variables in each model type. Finally, we present results from the sensitivity analyses. Figure 1 provides a graphical overview of the experimental setup, methods, and key findings from our analyses.

Between 2013 and 2021, there were 17,689 patients with a TAVI intervention in one of the 16 Dutch hospitals (labeled A–P). In total, 1,028 patients lacked an outcome measurement; therefore, these patients were excluded from the analysis.

The final TAVI dataset consisted of 16,661 records with an average outcome prevalence of 30-day mortality of 3.4%. The prevalence ranged from 1.2% to 5.8% between hospitals, with an intra-quartile range (IQR) of 2.8%–3.9% (Supplementary Table S2). From the list of all 33 candidate predictor variables, only the variable of frailty status was excluded for having more than 30% of its records missing.

### 3.1 Model performance

Predictive performance results for each model across both internal validation (CV) and external validation (LCOA) are reported in the following.

Due to the lower volume of TAVI records and low outcome prevalence, fitting a hospital-local model failed in some iterations of the CV analysis. From the 10 folds during CV, a local model could not be fitted in 100% of folds in centers L and P, 90% of folds in center M, 70% in center I, 60% in center K, 40% in N, 20% in C and J, and 10% in centers F and O (Supplementary Table S3).

#### 3.1.1 Discrimination

##### 3.1.1.1 Cross-validation

**Figure 2:**
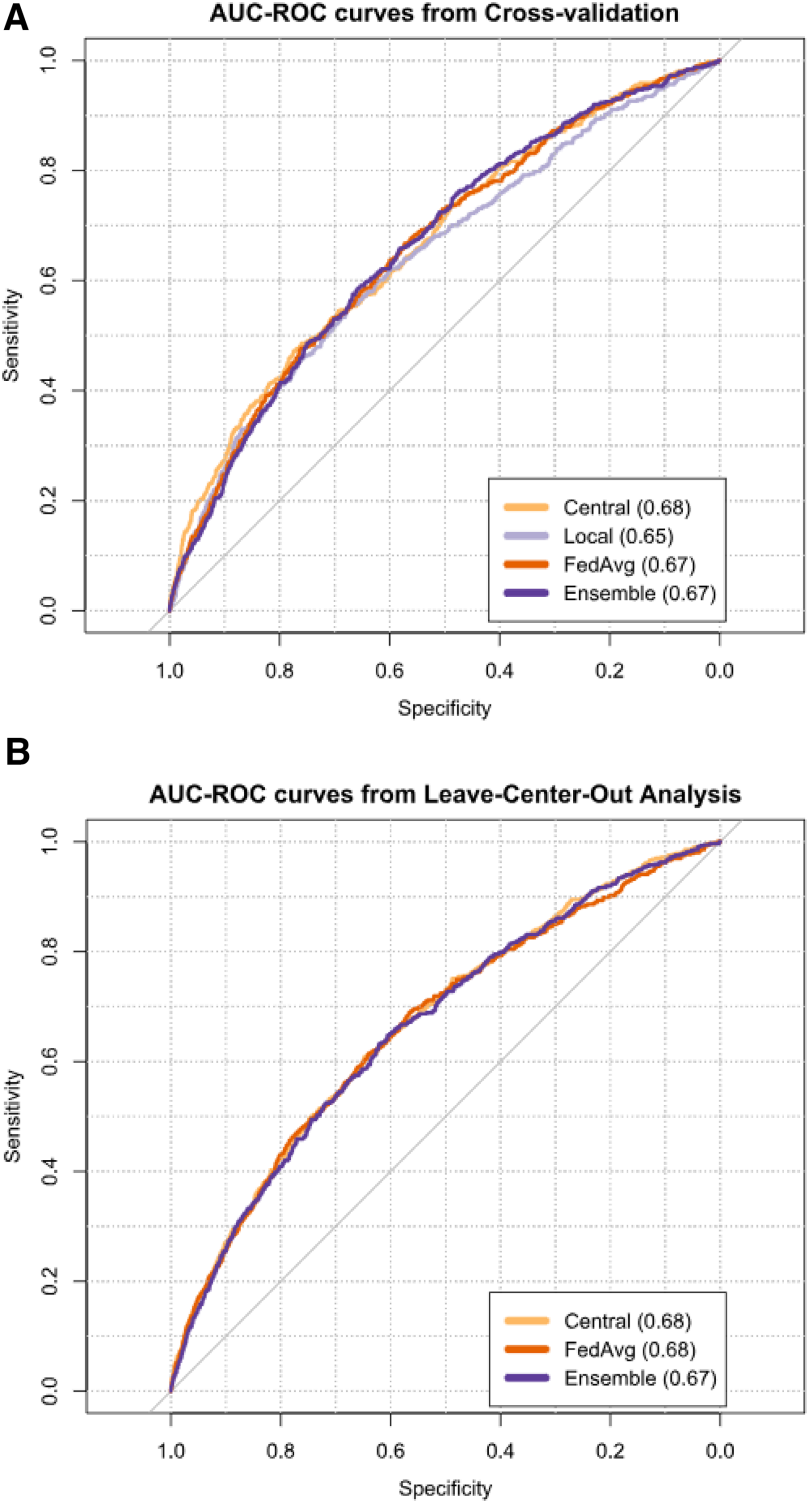
AUCs from cross-validation (**A**) and leave-center-out analysis (**B**) of TAVI patient 30-day mortality risk prediction models. Next to each model's name, its mean AUC is given.

*The central* model had the highest mean AUC during internal validation (0.68, 95% CI: 0.66–0.70), followed by *FedAvg* (0.67, 95% CI: 0.65–0.68), *ensemble* (0.67, 95% CI: 0.66–0.68), and *local* (0.65, 95% CI: 0.63–0.67) (Figure 2A, Supplementary Table S4). Comparing model AUCs for significant differences with the bootstrap method showed *central* to outperform *local* and *FedAvg* in two (20%) and 1 (10%) out of 10 folds, respectively (Supplementary Table S5). AUC results of local models ranged from 0.52 to 0.84 across centers (Supplementary Table S6).

##### 3.1.1.2 Leave-center-out

The meta-analysis pooled mean AUC from the LCOA was 0.68 (95% CI: 0.66–0.70) for the *central* model (Figure 2B, Supplementary Table S4), and AUC values ranged from 0.62 to 0.76 between external centers (Supplementary Table S7). *FedAvg* also had a mean AUC of 0.68 (95% CI: 0.65–0.70), and its AUC values for the individual centers ranged from 0.56 to 0.80. For the *ensemble*, the mean AUC was 0.67 (95% CI: 0.65–0.70), and AUC values ranged from 0.46 to 0.76 between external hospitals.

Bootstrap AUC testing from LCOA showed that both *FedAvg* and *ensemble* outperformed *central* in one hospital (P) (Supplementary Table S8). In another two centers (C and N), *FedAvg* outperformed *ensemble*, and in one hospital (H), *central* outperformed *ensemble*.

#### 3.1.2 Calibration

Calibration performance results varied across the different models and validation strategies.

Calibration graphs showed that all models suffered from over-prediction in the higher-risk ranges. To better inspect the lower-risk probabilities (found in the majority of the records), calibration graphs for a model were visualized excluding the top 2.5% of highest predicted probabilities.

##### 3.1.2.1 Cross-validation

**Figure 3:**
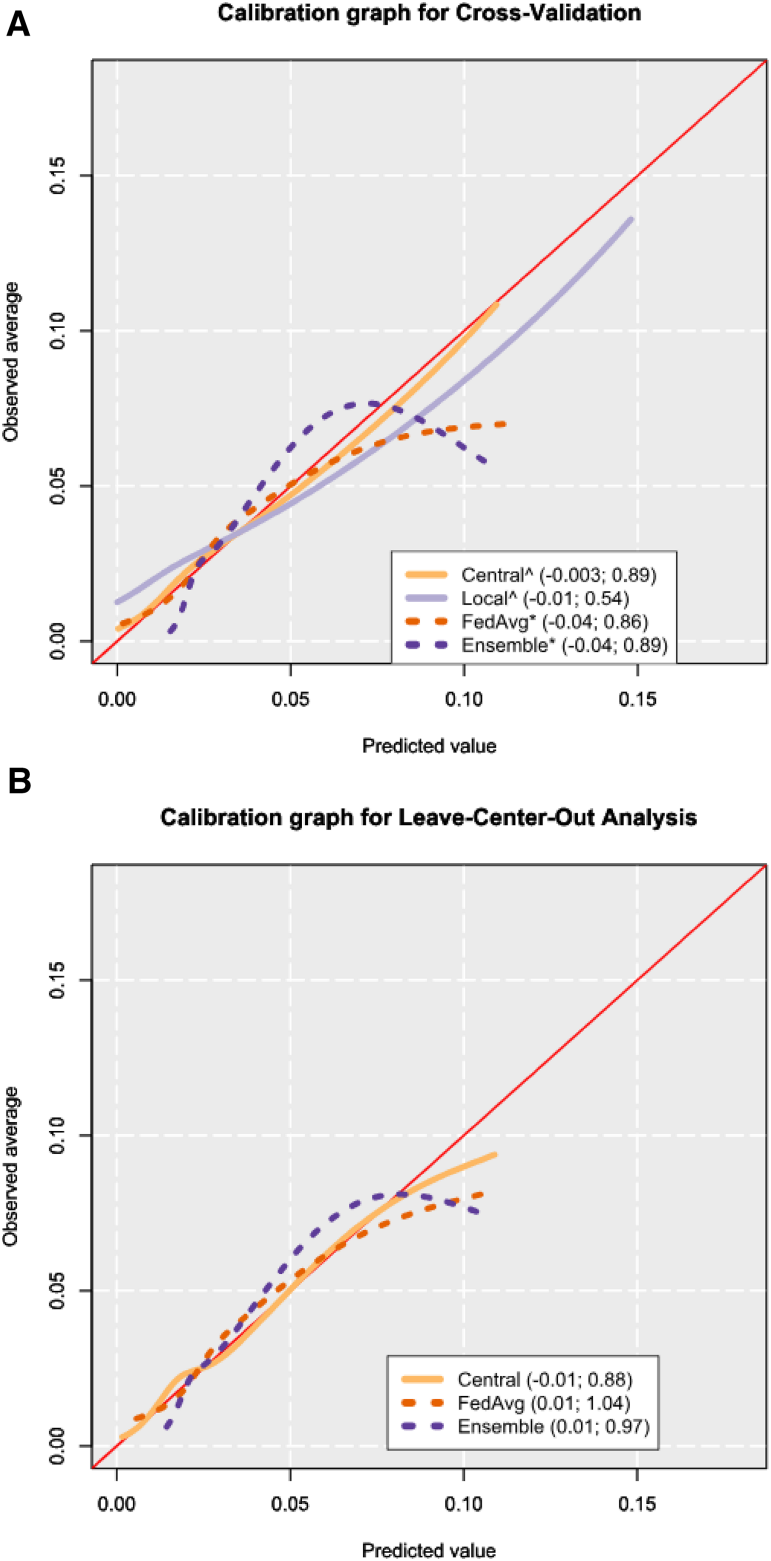
Calibration graphs from cross-validation (**A**) and leave-center-out analysis (**B**) results. The calibration graphs are shown after trimming the 2.5% highest predicted probabilities to focus on the bulk of the sample. The legend shows the calibration intercept and slope of each model, respectively, as obtained from the Cox method (16). Mean values for cross-validation were obtained by computing performance metrics on the combined predictions from all corresponding test sets. An asterisk (*) is placed after the names of the models where miscalibration was detected by way of the Cox method, and a hat (^) symbol is placed if miscalibration occurred in the calibration slope. The calibration intercept and slope values shown in the legend are calculated from all the predictions, including the 2.5% highest predicted probabilities.

From CV, all models showed miscalibration by slope when evaluated via the Cox method, and only *ensemble* showed miscalibration from its intercept (Figure 3A, Supplementary Table S9). *Central* had a calibration intercept of −0.003 (95% CI: −0.03 to 0.02) and a calibration slope of 0.89 (95% CI: 0.80–0.98). *Local* models had a mean calibration intercept in CV of −0.01 (95% CI: −0.04 to 0.01) but had a poor calibration slope of 0.54 (95% CI: 0.40–0.67). From the 14 hospitals where local models could be fitted (88% of all the hospitals in the dataset), miscalibration occurred in 13 of them (93%) (Supplementary Table S10). For *FedAvg*, the calibration intercept was −0.04 (95% CI: −0.07 to −0.02), and the calibration slope was 0.86 (95% CI: 0.78–0.93). *Ensemble* models showed a calibration intercept of −0.04 (95% CI: −0.06 to −0.01) and a calibration slope of 0.89 (95% CI: 0.82–0.96). Calibration graphs of the four models showed *central* to most closely resemble the ideal calibration graph, followed by *local* (Figure 3A).

##### 3.1.2.2 Leave-center-out

In the LCOA, the mean meta-analysis pooled calibration intercept for the *central* model was −0.01 (95% CI: −0.16 to 0.15) and the calibration slope was 0.88 (95% CI: 0.76–1.01) (Supplementary Table S9). Miscalibration was detected in 44% of external hospital validations for the *central* model (Supplementary Table S1^1^). In *FedAvg*, the calibration intercept was 0.01 (95% CI: −0.16 to 0.18), the calibration slope was 1.04 (95% CI: 0.89–1.19), and miscalibration occurred in 44% of the external hospitals. The *ensemble* model had a calibration intercept of 0.01 (95% CI: −0.14 to 0.16), the calibration slope was 0.97 (95% CI: 0.82–1.12), and miscalibration was seen in 38% of centers.

Similar to the calibration graph from CV, the calibration graph in LCOA showed *central* to most closely follow the line of the ideal calibration graph (Figure 3B).

#### 3.1.3 Net reclassification improvement

NRI comparison results showed *central* models to outperform the rest in predicting positive outcomes during CV and LCOA. From CV, *local* models were superior to the rest for predicting negative outcomes, while during LCOA, *central* models showed a higher NRI than the rest for negative outcomes. In both CV and LCOA, *FedAvg* beat *ensemble* in the case-negative group. In the LCOA case-positive group, *ensemble* had a better NRI than *FedAvg*. Full results from comparing model predictions using NRI can be found in Supplementary Results S1.

### 3.2 Predictors selected

From the final models fitted using the whole dataset, *FedAvg* and *ensemble* both used the same set of 20 variables (Supplementary Tables S12, S13).

The hospital-*local* models used between 2 and 14 variables (IQR 4–9). Selected variables occurring in at least 50% of all *local* models were age, left ventricular ejection fraction (LVEF), body mass index (BMI), BSA, and procedure access route. In the case of two hospitals (L and P), no *local* model could be trained due to insufficient TAVI record volumes with a positive outcome.

The *central* model selected 19 predictor variables (Supplementary Table S12), which comprised 13 predictors already selected by the other strategies, plus an additional 6 new predictors [Canadian Cardiovascular Society (CCS) class IV angina, critical preoperative state, dialysis, previous aortic valve surgery, previous permanent pacemaker, and recent myocardial infarction]. More information on the considered and selected variables can be found in Supplementary Table S14.

### 3.3 Sensitivity analyses

In such an analysis, where the recalibration step from model training was skipped for *FedAvg* and *ensemble* models, we saw that both performed significantly worse in calibration but not in AUC. In the second sensitivity analysis, where three low-volume heart centers (P, O, and N) were excluded from the analysis, the performance of the *ensemble* model remained mostly unchanged, while AUC was negatively affected for the other models. From this same analysis, an improvement was observed in calibration during CV for *FedAvg* and a worsening for *central* was observed during LCOA. Full results from the two sensitivity analyses are available in Supplementary Results S2.

## 4 Discussion

### 4.1 Summary of findings

In this study, we investigated the performance of two FL approaches compared to *central* and *local* approaches for predicting early mortality in TAVI patients. We showed that *FedAvg* and *ensemble* models performed similarly compared to a *central* model. The hospital-*local* models were worse in terms of average AUC compared to the other approaches.

Testing for AUC differences showed the *central* model to outperform *local* and *FedAvg* models but not *ensemble* during internal validation. The *local* models, however, did not significantly outperform the federated ones, suggesting that the AUC performance of *FedAvg* and *ensemble* lied somewhere between that of the *central* and *local* models.

*Central* and federated models performed similarly well in terms of calibration, whereas *local* model predictions were more frequently miscalibrated. Furthermore, in two cases, the *local* models could not be fitted due to the low number of positive outcome records in their datasets. Although *local* models were calibrated by design to their corresponding hospital-*local* training datasets (Table 1), this was often not sufficient to produce a good calibration on their corresponding test sets. While the federated models may not have been calibrated by design, they offered more options for recalibration (such as global, local, or federated recalibration). This could provide model developers with more fine-grained control over tradeoffs between maintaining data privacy and improving model calibration.

In the main experiments, the choice was made to use the *central* recalibration strategy (as opposed to *local* or federated) for the federated approaches. Although this approach requires the sharing of patient outcome data and model predictions between centers, it does offer the most promising recalibration approach of the three options.

In terms of NRI, there was an observed improvement from *local* to *FedAvg* and *ensemble* to *central* when looking at the outcome-positive group of records during internal validation (Supplementary Results S1).

When comparing the two federated approaches, it is difficult to say that one strategy was better than the other, as both had strengths and weaknesses. In terms of discrimination, *FedAvg* seemed to be slightly superior to the *ensemble* model. For model calibration during internal validation, *FedAvg* and *ensemble* showed near-identical results; however, in the external validation, the *ensemble* approach was miscalibrated in fewer external hospitals.

From an interpretability standpoint, the *FedAvg* model would be preferred to the *ensemble* one, as it delivers a single parametric model with predictor variables and their coefficients. The *ensemble*, on the other hand would, comprise a list of parametric models (which may not all use the same variables), plus a top-level parametric model that combines the outputs from the aforementioned list. While the *ensemble* model is not as easily interpretable immediately, techniques like metamodeling could be useful to bridge this gap (19).

It is worth noting that, although easily interpretable, the *FedAvg* model was more costly to develop than the *ensemble* one regarding computing resources. Depending on the number of hyperparameter values considered, we saw that the training times for the *FedAvg* model could easily become orders of magnitude larger than those for the *ensemble* model. In the current experiments, we developed our in-house frameworks for both federated approaches and encountered more hurdles with the *FedAvg* strategy—these included issues such as model convergence problems and the need to use a more elaborate variable selection strategy, which introduced the need for an additional hyperparameter.

### 4.2 Strengths and limitations

Our study has several strengths. It is the first study on employing federated learning in the TAVI population and one of the very few FL studies in cardiology. It is also based on a large national registry dataset consisting of all 16 hospitals performing TAVI interventions in the Netherlands. In addition, we provided a framework (in Table 1) of the various important elements to consider when adopting FL strategies in this context. We also considered multiple aspects of predictive performance and employed two validation strategies to prevent overfitting and optimism in the results. Finally, two sensitivity analyses were conducted to understand the robustness of our findings.

Our research also has limitations. We looked at FL prediction models for TAVI patients, considering only one outcome: the 30-day mortality. However, early post-operative mortality is a relevant and important clinical outcome in the TAVI patient group.

From a privacy perspective of local hospitals, we did not evaluate additional techniques that could be used to preserve patient privacy at local centers (such as differential privacy).

We also considered only one type of ensemble approach (mean volume-weighted ensemble) and only one type of federated aggregation approach (*FedAvg*),although a number of alternatives are available in both cases (11, 20). Although relatively small, the group of patients excluded from the analysis due to missing outcome values could have somewhat biased our results in model performance. Changes over time in TAVI intervention modalities and patient selection protocols could also have impacted model performance estimates (21).

Finally, we did not extensively tune hyperparameters, which might have affected the performances of the *FedAvg* and *ensemble* models (22).

### 4.3 Comparison with literature

Few studies have investigated the impact of FL in the cardiology domain (23–25). In only one study, the authors look at risk models for TAVI patients (23). In this study, Lopes et al. developed non-parametric models for predicting 1-year mortality after TAVI on a dataset from two hospitals. They compared hospital-local model performance against that of federated ensemble models and found the ensemble models to outperform the local ones. Our findings on the ensemble model's superior performance align with the study by Lopes et al. However, we expanded on their findings, first, by evaluating predictive performance with a much larger number of hospitals (16 vs. 2); second, by considering model calibration performance and NRI in addition to AUC; third, by performing additional geographic validation; and finally by considering a centralized model strategy as a baseline in addition to local and federated ones.

Another study by Goto et al. looked at training FL models to detect hypertrophic cardiomyopathy using ECG and echocardiogram data from three hospitals (24). The authors considered the AUC metric for discrimination and looked at *FedAvg* and local hospital models. They reported that the FL models outperform local models in terms of AUC, something we also observed in the current study.

In other medical domains, FL models have previously been evaluated on their performance compared to models derived from non-FL techniques.

A similar study to ours that described the benefits of using centralized models compared to federated and local ones is that by Vaid et al. (26). In their study, the authors developed prediction models for COVID-19 patient 7-day mortality outcomes and reported that in five out of five hospital datasets, the models derived from a central development strategy outperformed both local and federated models in terms of AUC. This finding was corroborated in our study for the local models but not for the federated models, which performed on par with the central ones. Differences in the domain of application and in the datasets may explain this. From inspecting the NRI of our models, however, it became clear that the central models offered an improvement on the federated ones, albeit not a statistically significant one. The findings from Vaid et al. (26), namely, that local models tended to underperform compared to central and federated models (in AUC but also in calibration), align with our findings.

Sadilek et al. (2) looked at eight previous studies of prediction models that used a centralized model approach and attempted to reproduce these eight models with the modification of using FL in their development strategies. From the eight models they evaluated, only one looked at hospital as the unit of the federation and reported a coefficient estimate for extrapulmonary tuberculosis in individuals with HIV. This coefficient differed significantly between the centralized and federated approaches. However, in a different setting, we observed similar findings with respect to the coefficients of our federated and centralized TAVI risk models.

### 4.4 Implications and future studies

For clinicians wanting to adopt a federated learning approach for developing prediction models for TAVI patients, our recommendation would be to use the *ensemble* strategy if predictive performance is most important, while the *FedAvg* strategy should be considered if one is willing to sacrifice a bit of model performance for better interpretability.

From the federated learning aspects overview (Table 1), possible model strategy setup options were described. While we attempted to make a comprehensive experimental setup, the purpose of this study was not to evaluate all possible options from this table. This methods’ overview could thus be further used to guide an evaluation of how predictive performance would change if one explored the various setup options.

Further studies should be done to refine the *FedAvg* and *ensemble* models, focusing on the use of additional techniques to enhance privacy-preservation and hyperparameter tuning (22). The evaluation of model performance should also be considered for other outcomes in addition to the 30-day post-operative mortality, as well as for other FL models in addition to the two types considered here. Future research could also investigate further aspects of model predictive performance by incorporating additional metrics, such as model's sharpness, the area under the precision-recall curve (AUC-PR), and the F1 score. In addition, the questions of investigating model performance in terms of scalability and computing resource requirements are important and merit future research.

The limitation of fitting a *local* model in centers with an insufficient number of case records emphasizes an issue that has not been extensively covered. This area represents a potential direction for future research to improve predictive modeling in such contexts.

Our experiments focused on parametric models, or more precisely models that use logistic regression. It is unclear whether the current findings would translate into federated learning for non-parametric or deep learning models.

Performance variations observed across different models emphasize the importance of selecting the appropriate model development strategy for each individual setting. Finally, examining the potential benefits and limitations of federated learning in cardiology, in general, merits future research.

## 5 Conclusion

Both the *FedAvg* and *ensemble* federated learning models had comparable AUC and calibration performance to the *central* risk prediction model of TAVI patients. This suggests the *FedAvg* and *ensemble* models are strong alternatives to the *central* model, emphasizing their potential effectiveness in the multicenter dataset.

The heterogeneity in performance across different hospitals underscores the importance of local context and sample size. Future research should further explore and enhance these distributed learning methods, particularly focusing on the robustness of federated learning models across diverse clinical settings.

## Data Availability

The data analyzed in this study is subject to the following licenses/restrictions: permission to use the data underlying this article was granted by the Netherlands Heart Registration (NHR). Permission to reuse the data can be addressed to the NHR (https://nhr.nl/wetenschappelijk-onderzoek/#aanvragen). Requests to access these datasets should be directed to https://nhr.nl/wetenschappelijk-onderzoek/#aanvragen.

## Supplementary material

The Supplementary Material for this article can be found online at: https://www.frontiersin.org/articles/10.3389/fcvm.2024.1399138/full #supplementary-material.
