# Supplementary material for: Performance of federated learning-based models in the Dutch TAVI population was comparable to central strategies and outperformed local strategies
Source: Front Cardiovasc Med. 2024 Jul 5;11:1399138. doi: 10.3389/fcvm.2024.1399138 (PMC11257923; doi:10.3389/fcvm.2024.1399138)
Supplement: Supplementary file 1 [file Datasheet1.pdf]

Supplementary Material

1 Appendix A: Transcatheter Heart Implantation Committee Members

The following physicians are the members of the Transcatheter Heart Implantation (THI) Registration Committee of the NHR. They represent the hospitals that have provided data for the THI registry.

Dr.	B.J.L.	van den	Branden	Amphia
Dr.	MV		Vis	Amsterdam UMC, locatie AMC
Dr.	W.A.L.		Tonino	Catharina Ziekenhuis
Prof. dr.	NMDA	van	Mieghem	Erasmus MC
Dhr.	C.E.		Schotborgh	HagaZiekenhuis
Dr.	R.S.		Hermanides	Isala
Dhr.	F.	van der	Kley	Leids Universitair Medisch Centrum
Dr.	P.		Vriesendorp	Maastricht UMC+
Dhr.	D.		Stecher	Medisch Centrum Leeuwarden
Dr.	M.G.		Stoel	Medisch Spectrum Twente
Dr.	G.		Amoroso	OLVG
Mw.	M	van	Wely	Radboudumc
Dr.	L		Timmers	St. Antonius Ziekenhuis
Dr.	M		Voskuil	UMC Utrecht
Dhr.	H.W.	van der	Werf	Universitair Medisch Centrum Groningen

2 Appendix B: Supplementary Figures

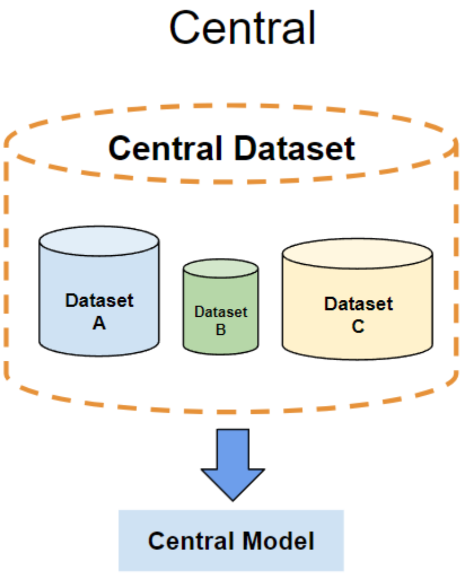

Supplementary Figure 1. Diagram of the Central model development strategy.
In the *Central* model development strategy the datasets from all hospitals are first combined into one single centralized dataset. Using this combined dataset from all hospitals, a prediction model is then fitted, and later used for making predictions.

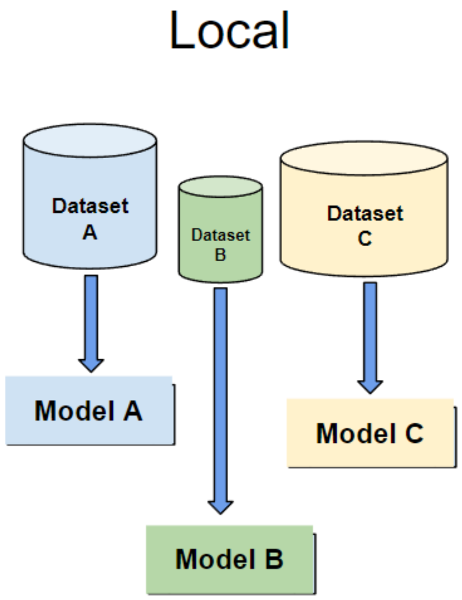

Supplementary Figure 2. Diagram of the Local model development strategy.
In the *Local* model development strategy, a local model is fit on each center's local dataset. Each hospital-local model is then used for making predictions only on patients belonging to the model's corresponding local hospital.

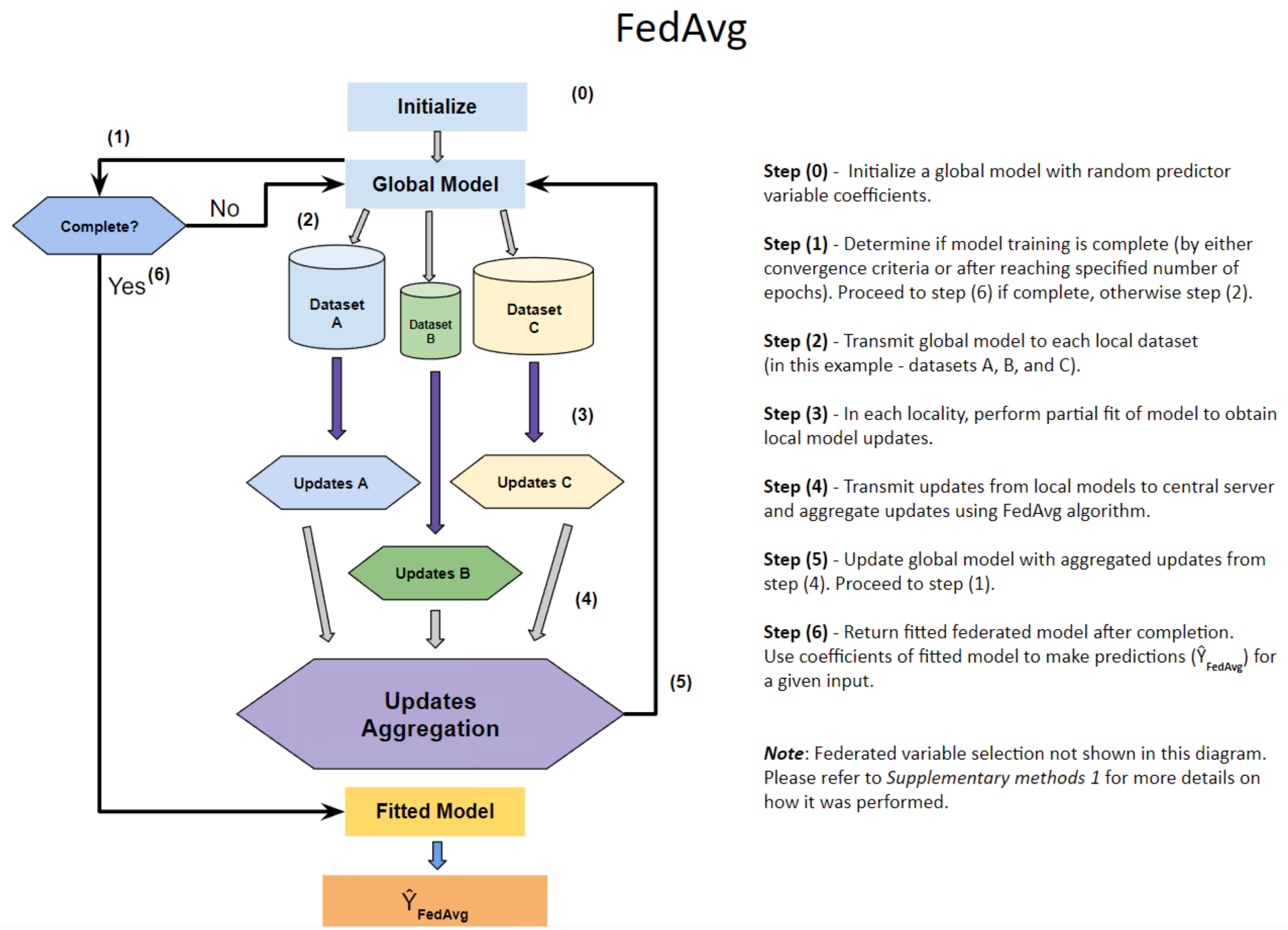

Supplementary Figure 3. Diagram of FedAvg model development strategy.

In the *FedAvg* method, each participating center would train a local model using its own data, and its local variable selection (which was done before the start of the training), for one epoch, and share its model parameters with a central server (1). The central server then aggregates the parameters from the local models to produce a global model (Supplementary Figure 3). This global model is then sent back to the individual hospitals for further local training in the next epoch. This iterative process continues until either convergence or a pre-specified number of epochs is reached.

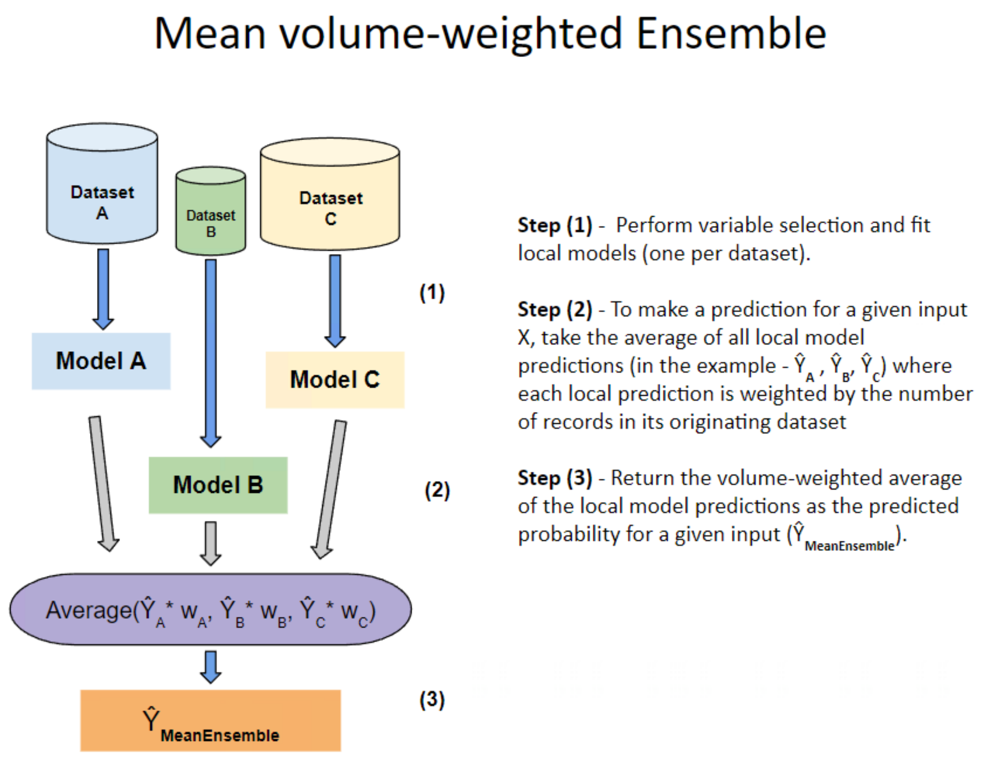

Supplementary Figure 4. Diagram of mean volume-weighted ensemble model development strategy.

In the *ensemble* model strategy, a local model is fit on each center's data. Each local model would then make predictions for the records across all centers. This would produce a number of predictions (16 in our case, one per local model) for each record in the dataset. The predicted output of this model strategy is then formed by aggregating the predictions of each local model. With this strategy, only model predictions are transmitted between hospitals. Aggregation of local model predictions is then done by record-volume weighted averaging across hospitals. For example, suppose we had a setup with only two hospitals, A and B, where hospital A had 500 records and hospital B had 1000 records. For a given new record, if the local model from A predicted a risk of 0.5 and the model from B predicted a risk of 0.75, then the final prediction from the ensemble model would be (500/1500)*0.5 + (1000/1500)*0.75 = 0.67.

3 Appendix C: Supplementary Tables

Supplementary Table 1. Definitions of candidate predictor variables.

Variable	Definition
**Age (years)**	Age of patient at time of intervention measured in years.
**Body surface area BSA (m^2)**	Estimated from patient height and weight using the Mosteller formula [31] BSA = sqrt([Height (cm) x Weight (kg)] / 3600); The following threshold values were used for summarizing body surface area (BSA) in Supplementary Table 2 - Women 18 years: 1.726m2, 19-79 years: 1.830m2, 80+ years: 1.638m2; Men 18 years: 1.980m2, 19-79 years: 2.060m2, 80+ years: 1.920m2. Threshold values were obtained from (2).
**Body mass index BMI (kg/m^2)**	Estimated from patient height and weight using the standard formula BMI = Weight (kg) /[Height (m) * Height (m)]
**Serum creatinine (mmol/L)**	The last preoperatively determined blood creatinine concentration in micromol per liter (µmol/l), measured no more than 3 months ago before the current intervention.
**Estimated glomerular filtration rate eGFR (mL/min/1.73m²)**	If gender male: 175*((SK/88.4)^- 1.154)*(Age^-0.203) If sex female: (175*((SK/88.4)^-1.154)*(Age^-0.203))*0.742; SK = Serum creatinine (mmol/L); Age = Age (years)
**Left Ventricular Ejection Fraction LVEF**	The percentage of the end-systolic volume of blood in the left ventricle to the end-diastolic volume, or relative stroke volume. Use the most recent pre-intervention determination documented in a diagnostic report.
**Systolic Pulmonary Artery Pressure (mmHg)**	The percentage of the end-systolic volume of blood in the left ventricle to the end-diastolic volume, or relative stroke volume. Using the most recent pre-intervention determination documented in a diagnostic report.
**Sex**	Male (denoted by value 0) or Female (denoted by value 1).
**Diabetes Mellitus DM**	The status of Diabetes Mellitus for a patient expressed in the following categories: 1) no diabetes; 2) diabetes without treatment; 3) diabetes with treatment; 4) diabetes with unknown treatment
**Chronic lung disease**	Indicated by long-term use of bronchodilators or steroids because of lung disease.
**Extra-cardiac arteriopathy**	If one or more of the criteria below applies: Intermittent claudication; Carotid occlusion or > 50% stenosis; Amputation due to arterial disease; Previous or planned surgery on abdominal aorta, arteries of the limbs or carotids.
**Neurological dysfunction**	Disease that severely limits ambulatory or daily functioning.
**Previous cardiac surgery**	Previous cardiac surgery, involving opening of the pericardium. This also includes transapical transcatheter heart interventions.
**Critical preoperative state**	If there is one or more of the criteria below: Preoperative ventricular tachycardia or fibrillation at the start of the ;intervention, preoperative sudden death survivor or preoperative ;Resuscitation ;Pre-operative ventilation before arrival in the operating room Pre-operative administration of inotropics ;IABP inserted preoperatively ;Preoperative renal failure (anuria or oliguria < 10 ml/hr).
**Recent myocardial infarction**	Having had a myocardial infarction in the 90 days prior to the current intervention.

**Dialysis**	Hemodialysis or peritoneal dialysis for renal failure on a continuous basis at the time of admission for the current intervention. This also includes continuous venovenous haemofiltration due to renal failure (not if this is temporary to only remove fluid for heart failure).
**Poor mobility**	Severe impairment of mobility secondary to musculoskeletal or neurological dysfunction.
**Functional New York Heart Association (NYHA) class**	The New York Heart Association (NYHA) functional classification class for heart failure. The highest class in the period of 2 weeks before the intervention.
**Canadian Cardiovascular Society (CCS) class IV angina**	The patient has angina pectoris according to Class IV of the Canadian Cardiovascular Classification System (CCS).
**Procedure acuity**	Status of the current intervention divided into:; Elective: Routine recording before surgery; Urgent: Patients who were not electively admitted for surgery but; require an intervention for medical reasons within the current; intake. These patients cannot be sent home without a; final procedure; Emergency: Unplanned intervention that occurs after the decision to operate to; medical reasons cannot wait until the start of the next working day; Rescue: Patients undergoing cardiopulmonary resuscitation (external cardiac; massage) on the way to the operating room or prior to; the administration of anesthesia. This does not apply to cardiopulmonary; resuscitation following the administration of anesthesia.
**Previous Cerebrovascular Accident (CVA)**	A neurologist has determined that a postoperative stroke during the hospitalization of the current intervention has occurred (excluding TIA). CVA = permanent neurological dysfunction diagnosed by a neurologist as due to focal ischemia of the brain, spinal cord, or retina, caused by an acute infarction of the neurological tissue due to thrombosis, embolism, systemic hypoperfusion or bleeding.
**Previous aortic valve surgery**	Having performed an aortic valve intervention prior to the current intervention. This concerns both replacements and plastics, and conventional as transcatheter valve interventions (TAVI) to the aortic valve.
**Previous permanent pacemaker**	Having a permanent pacemaker implant performed prior to the current intervention, independent of functionality.
**Anesthesia**	Performing general anesthesia for the current intervention. It This does not include allowing the patient to sleep lightly (sedation).
**Balloon pre-TAVI**	The occurrence of balloon predilation of the aortic valve to be replaced during the current intervention.
**Percutaneous Aortic Balloon Valvuloplasty (PABV)**	PABV (Percutaneous Aortic Balloon Valvuloplasty) dilatation of the aortic valve implant after the last implant performed during the current intervention.
**Access route**	The most invasive access route used to get the last insertion aortic valve implant through a catheter (access site) during the current intervention. Transfemoral; Transfemoral-Percutaneous; Via subclavian artery; Transapical; Direct transaortic.
**Frailty status category**	Measured using the Edmonton Frail Scale prior to the current intervention. The score per individual question must be registered.

**Procedure weight (2 operations)**	The weight that can be assigned to the current planned intervention on; based on major interventions, in which the following categories apply: Isolated CABG; Intervention consisting of only a CABG and no other cardiac major; surgery; 1 procedure (no CABG); Intervention consisting of 1 major procedure that is not a CABG; Examples: single valve surgery, ascending aorta replacement, or; correction of septal defect, etc; 2 surgeries; Intervention consisting of 2 major procedures. Examples: CABG +; Aortic valve surgery, CABG + MVR, Aortic valve surgery + aortic replacement; ascendens, CABG + Maze, Aortic valve surgery + MVR, etc; 3 or more surgeries; Intervention consisting of 3 or more major procedures. Examples:; Aortic valve surgery + MVR + CABG, MVR + CABG + annuloplasty; tricuspid valve, aortic root replacement with aortic valve surgery or plastic; + coronary reimplantation + root and ascending replacement, etc; Only major cardiac procedures are included in the total. Examples of interventions; that are not considered major are: sternotomy, closure of sternum,; myocardial biopsy, insertion of IABP, leads, additional pacemaker placement; and/or ICD, closure of aortotomy, closure of atriotomy, atrial appendage,; coronary endarterectomy as part of a CABG, etc.
**Unstable angina pectoris**	Angina pectoris requiring intravenous nitrate therapy up to and including the arrival at the operating room to perform the current intervention
**Thoracic aortic surgery**	Surgical intervention on ascending, arch or descending aorta during the current intervention.
**Post-MI Ventricular Septal Rupture (VSR)**	Current surgery is being performed because of a post-infarction ventricular septal rupture (VSR) defined as a defect in the interventricular septum due to rupture of myocardial infarction.
**Endocarditis**	At the time of intervention, the patient is still being treated with an antibiotic for endocarditis.

Supplementary Table 2. Baseline characteristics of TAVI patients from the Netherlands Heart Registration (NHR) for 16 Dutch hospitals from the period Jan 1st 2013 – Dec 31st 2021.

center	ALL	A	B	C	D	E	F	G	H	I	J	K	L	M	N	O	P
**TAVI N (%)**	**16661 (100)**	**2047 (12.3)**	**1592 (9.6)**	**1575 (9.5)**	**1517 (9.1)**	**1244 (7.5)**	**1132 (6.8)**	**1081 (6.5)**	**1170 (7.0)**	**1044 (6.3)**	**1012 (6.1)**	**922 (5.5)**	**636 (3.8)**	**660 (4.0)**	**553 (3.3)**	**313 (1.9)**	**163 (1.0)**
**30-day-mortality (%)**	3.4	2.9	3.7	3.9	4.5	3.0	3.4	3.1	3.4	2.7	4.1	2.8	1.6	2.6	4.3	5.8	1.2
**Age years (mean)**	79.6	79.6	78.5	79.8	80.1	80.7	79.7	78.7	78.5	80.3	78.8	80.6	80.5	79.7	80.8	78.8	80.6
**LVEF (mean)**	50.3	48.7	52.6	50.3	50.3	48.9	49.5	52.5	53.2	51.9	49.1	49.5	49.2	49.4	49.2	49.2	50.5
**sPAP mmHg (mean)**	29.9	29.2	36.8	29.1	29.8	38	29.6	27.2	29.4	26.0	30.9	27.3	29	29.2	29.1	29.6	28.2
**NYHA class 3 (%)**	52.0	53.4	49.7	43.6	57.5	60.8	45	26.5	41.7	51.7	76.0	53.7	53.2	44.1	59.9	52.4	53.8
**NYHA class 4 (%)**	6.5	7.0	10.1	8.2	7.9	2.7	2.5	4.4	3.0	6.7	9.5	3.0	2.8	4.1	8.6	26.5	2.1
**Access route transfemoral (%)**	82.2	0.5	9.0	5.2	5.0	7.8	4.1	3.4	6.9	2.5	3.6	1.7	8.9	5.2	9.5	3.1	6.0
**Access route direct aortic (%)**	6.3	0.0	0.1	0.0	0.6	0.1	1.3	0.0	0.1	0.7	0.0	0.0	0.0	0.6	2.4	0.0	0.3
**BMI (mean)**	27.2	27.0	27.3	27.0	26.5	27.5	26.7	27.4	27.8	27.5	27.9	27.6	27.2	26.8	27.4	26.5	26.8
**BSA threshold (%) ***	42.3	41.5	41.5	40.1	41.5	43.2	43.0	40.6	44.3	45.3	47.5	44.6	42.8	44.8	47.9	40.1	43.2
**sCreat mmol/L (mean)**	104.6	104.4	108.7	107.7	109.2	100.2	103.9	106.4	99.6	109.1	99.5	99.3	104.7	99.3	101.5	112.2	108.9
**eGFR (mean)**	60.9	62.0	60.7	58.8	59.3	62.2	62.8	58.8	63.7	56.5	61.3	62.3	60.1	64.8	60.6	60.6	57.9
**Diabetes Mellitus (%)**	27.1	25.0	31.4	26.7	25.6	28.6	27.7	25.3	30.6	29.8	26.6	25.6	28.7	23.0	22.6	25.2	19.3
**Sex (Female %)**	49.2	43.6	46.5	49.0	47.9	48.7	52.3	50.8	48.5	52.4	48.0	46.6	46.2	53.2	50.9	47.5	52.3

TAVI - Transcatheter Aortic Valve Implantation,
LVEF - Left Ventricular Ejection Fraction,
sPAP - Systolic Pulmonary Artery Pressure,
NYHA - New York Heart Association classification,
BMI - Body Mass Index,
BSA - Body Surface Area,
sCreat - Serum Creatinine,
eGFR - Estimated Glomerular Filtration Rate,
DM - Diabetes Mellitus.
*- patients with BSA greater than defined threshold- Women 18 years: 1.726 m^2, 19-79 years: 1.830 m^2, 80+ years: 1.638 m^2; Men 18 years: 1.980 m^2, 19-79 years: 2.060 m^2, 80+ years: 1.920 m^2 .

Supplementary Table 3. Summary of failed local model derivation occurrences during Cross-Validation of 30-day TAVI post-operative mortality risk prediction models.

Center	A	B	C	D	E	F	G	H
% folds	0%	0%	20%	0%	0%	10%	0%	0%
**Center**	**I**	**J**	**K**	**L**	**M**	**N**	**O**	**P**
% folds	70%	20%	60%	100%	90%	40%	10%	100%

Supplementary Table 4. AUC results from Cross-Validation and Leave-Center-Out Analysis of 30-day TAVI post-operative mortality risk prediction models from four model development strategies (*central*, *local*, *FedAvg*, *ensemble*).

Validation Strategy	Model Strategy	AUC Mean (95%CI)
Cross-validation	Central	0.68 (0.66-0.70)
	Local	0.65 (0.63-0.67)
	FedAvg	0.67 (0.65-0.68)
	Ensemble	0.67 (0.66-0.68)
Leave-Center-Out	Central	0.68 (0.66-0.70)
	FedAvg	0.68 (0.65-0.70)
	Ensemble	0.67 (0.65-0.70)

Supplementary Table 5. AUC bootstrap test results from 10-fold Cross-Validation of 30-day TAVI post-operative mortality risk prediction models from four model development strategies (*central*, *local*, *FedAvg*, *ensemble*). Shown are only folds wherein a difference in AUCs between models was detected.

Fold#	Outperformed	Underperformed
3	Central	Local
5	Central	Local
3	Central	FedAvg

AUC test was performed using bootstrap sampling with 3000 bootstrap samples (3).

Supplementary Table 6. Per center AUC results of *local* model development strategy from Cross-Validation (CV) of 30-day TAVI post-operative mortality risk prediction models.

Center	AUC (95%CI)	Center	AUC (95%CI)
**Pooled**	0.65 (0.63;0.67)	**I**	0.56 (0.38;0.75)
**A**	0.70 (0.63;0.77)	**J**	0.52 (0.42;0.62)
**B**	0.61 (0.53;0.69)	**K**	0.53 (0.31;0.74)
**C**	0.60 (0.52;0.69)	**L**	NA
**D**	0.70 (0.63;0.76)	**M**	0.84 (0.63;1.00)
**E**	0.60 (0.50;0.70)	**N**	0.67 (0.54;0.80)
**F**	0.60 (0.50;0.70)	**O**	0.72 (0.58;0.85)
**G**	0.65 (0.54;0.75)	**P**	NA
**H**	0.77 (0.71;0.83)		

Each center's AUC was calculated by averaging the AUC results of each fold from CV. The pooled mean AUC was calculated by random-effects meta-analysis pooling of all center-specific AUCs with the hospital as the random effect (excluding centers where no model could be fit).
NA = Not Applicable.

Supplementary Table 7. AUC results of three model development strategies (*central*, *FedAvg*, and *ensemble*) of 30-day TAVI post-operative mortality risk prediction models from Leave-Center-Out Analysis of 16 Dutch hospitals (A-P).

**Model Strategy**	**Central**	**FedAvg**	**Ensemble**
**Center**		AUC (95%CI)	
A	0.71 (0.65;0.78)	0.70 (0.63; 0.77)	0.72 (0.66;0.79)
B	0.69 (0.62;0.76)	0.66 (0.59; 0.73)	0.67 (0.60;0.74)
C	0.63 (0.56;0.71)	0.67 (0.60; 0.73)	0.60 (0.53;0.68)
D	0.69 (0.63;0.76)	0.72 (0.66; 0.79)	0.72 (0.66;0.78)
E	0.66 (0.56;0.75)	0.60 (0.51; 0.70)	0.66 (0.57;0.75)
F	0.70 (0.62;0.77)	0.66 (0.57; 0.75)	0.69 (0.60;0.77)
G	0.71 (0.61;0.81)	0.72 (0.63; 0.81)	0.74 (0.66;0.83)
H	0.66 (0.58;0.75)	0.65 (0.56; 0.74)	0.65 (0.56;0.74)
I	0.65 (0.54;0.76)	0.67 (0.56; 0.77)	0.69 (0.58;0.80)
J	0.62 (0.52;0.71)	0.61 (0.52; 0.71)	0.59 (0.51;0.68)
K	0.71 (0.60;0.81)	0.68 (0.57; 0.78)	0.73 (0.64;0.83)
L	0.73 (0.58;0.88)	0.74 (0.56; 0.93)	0.76 (0.63;0.88)
M	0.65 (0.53;0.78)	0.60 (0.47; 0.74)	0.65 (0.54;0.77)
N	0.76 (0.66;0.86)	0.79 (0.69; 0.88)	0.75 (0.65;0.85)
O	0.74 (0.61;0.87)	0.80 (0.70; 0.90)	0.73 (0.59;0.87)
P	0.63 (0.09;1.00)	0.56 (0.01; 1.00)	0.46 (0.00;1.00)

Supplementary Table 8. AUC bootstrap test results of 30-day TAVI post-operative mortality risk prediction models from three model development strategies (*central*, *FedAvg*, *ensemble*) from Leave-Center-Out analysis of 16 hospitals.

Center	Outperformed	Underperformed
C	FedAvg	Ensemble
H	Central	Ensemble
N	FedAvg	Ensemble
P	FedAvg	Central
P	Ensemble	Central

AUC test was performed using bootstrap sampling (nboot = 3000) (3).

Supplementary Table 9. Summary of mean model predictive performance results for calibration intercept and slope metrics of 30-day TAVI post-operative mortality risk prediction models from four model development strategies (*central*, *local*, *FedAvg*, *ensemble*).

**Validation Strategy**	**Calibration**	
**Cross-Validation**	Intercept (95%CI)	Slope (95%CI)
**Model Strategy**		
Central*	-0.003 (-0.03;0.02)	0.89 (0.80;0.98)
Local*	-0.01 (-0.04;0.01)	0.54 (0.40;0.67)
FedAvg*	-0.04 (-0.07;-0.02)	0.86 (0.78;0.93)
Ensemble*	-0.04 (-0.06;-0.01)	0.89 (0.82;0.96)
**Leave-Center-Out**		
**Model Strategy**		
Central	-0.01 (-0.16;0.15)	0.88 (0.76;1.01)
FedAvg	0.01 (-0.16;0.18)	1.04 (0.89;1.19)
Ensemble	0.01 (-0.14;0.16)	0.97 (0.82;1.12)

Calibration intercept and slope values were computed using the Cox approach (4).
Mean values for cross-validation were obtained by computing performance metrics on the combined predictions from all corresponding test sets. Mean values for the Leave-Center-Out analysis results were obtained by meta-analysis pooling the 16 external validation results (one per each hospital in the dataset). * - denotes detected model miscalibration in either intercept or slope by way of the Cox method (4).

Supplementary Table 10. Per center calibration intercept and slope results of *local* model development strategy from Cross-Validation (CV) of 30-day TAVI post-operative mortality risk prediction models.

Center	Calibration [mean (95%CI)]		Center	Calibration [mean (95%CI)]	
**Pooled**	Intercept	-0.01(-0.04;0.01)*	**I**	Intercept	-0.15(-0.86;0.56)*
	Slope	0.54(0.40;0.67)		Slope	-0.56(-2.05;0.66)
**A**	Intercept	0.00(-0.27;0.26)*	**J**	Intercept	0.01(-0.35;0.37)*
	Slope	0.69(0.43;0.96)		Slope	0.06(-0.42;0.52)
**B**	Intercept	-0.10(-0.37;0.17)*	**K**	Intercept	0.00(-0.64;0.63)*
	Slope	0.42(0.16;0.67)		Slope	0.07(-0.89;0.95)
**C**	Intercept	-0.02(-0.31;0.27)*	**L**	Intercept	NA
	Slope	0.50(0.05;0.95)		Slope	NA
**D**	Intercept	0.00(-0.25;0.25)*	**M**	Intercept	0.19(-1.22;1.61)*
	Slope	0.72(0.48;0.95)		Slope	-1.13(-2.91;0.49)
**E**	Intercept	0.00(-0.33;0.34)*	**N**	Intercept	-0.08(-0.64;0.48)*
	Slope	0.50(0.12;0.88)		Slope	0.43(-0.01;0.92)
**F**	Intercept	0.03(-0.32;0.38)*	**O**	Intercept	0.02(-0.51;0.56)*
	Slope	0.42(-0.02;0.87)		Slope	0.56(0.14;0.98)
**G**	Intercept	-0.12(-0.47;0.24)*	**P**	Intercept	NA
	Slope	0.21(-0.07;0.49)		Slope	NA
**H**	Intercept	-0.03(-0.36;0.30)			
	Slope	0.78(0.50;1.06)			

An asterisk '*' is placed next to the calibration intercept result of each model where miscalibration was detected by way of the Cox method (4).
Each center's calibration intercept and slope was calculated by averaging the calibration intercept and slope results of each fold from CV. The pooled mean calibration intercept and slope were calculated by random-effects meta-analysis pooling of all center-specific calibration intercepts and slopes with the hospital as the random effect (excluding centers where no model could be fit).
NA = Not Applicable.

Supplementary Table 11. Calibration intercept and slope results of three model development strategies (*Central*, *FedAvg*, and *Ensemble*) from Leave-Center-Out analysis (LCOA) of 16 Dutch hospitals (A-P) of 30-day TAVI post-operative mortality risk prediction models.

**Model Strategy**		**Central**	**FedAvg**	**Ensemble**
Center		**Calibration** [mean (95%CI)]		
**A**	Intercept	-0.28(-0.55;-0.02)*	-0.23 (-0.49; 0.03)	-0.21(-0.47;0.05)
	Slope	0.95(0.65;1.26)	0.87(0.57; 1.18)	1.00(0.68;1.31)
**B**	Intercept	0.23(-0.05;0.50)*	0.36 (0.10; 0.62)*	0.20(-0.06;0.47)
	Slope	0.64(0.37;0.89)	0.86(0.44; 1.26)	0.91(0.51;1.29)
**C**	Intercept	0.13(-0.13;0.39)	0.29 (0.03; 0.55)*	0.08(-0.18;0.34)*
	Slope	0.63(0.25;1.00)	1.04(0.56; 1.53)	0.52(0.09;0.90)
**D**	Intercept	0.41(0.16;0.66)*	0.38 (0.14; 0.63)*	0.34(0.10;0.59)*
	Slope	1.05(0.72;1.38)	1.41(1.00; 1.81)	1.11(0.76;1.45)
**E**	Intercept	-0.40(-0.74;-0.07)*	-0.23 (-0.56; 0.10)	-0.37(-0.71;-0.04)*
	Slope	0.79(0.36;1.19)	0.95(0.40; 1.46)	1.01(0.61;1.40)
**F**	Intercept	0.15(-0.17;0.48)	0.22 (-0.11; 0.55)	0.09(-0.24;0.42)
	Slope	0.89(0.50;1.26)	0.86(0.41; 1.27)	0.83(0.41;1.22)
**G**	Intercept	0.10(-0.25;0.44)	-0.06 (-0.40; 0.28)	0.10(-0.25;0.44)
	Slope	1.22(0.81;1.64)	1.26(0.76; 1.75)	1.35(0.82;1.90)
**H**	Intercept	-0.10(-0.42;0.22)	-0.04 (-0.35; 0.28)	-0.06(-0.38;0.26)
	Slope	0.81(0.38;1.23)	0.93(0.38; 1.47)	0.70(0.17;1.19)
**I**	Intercept	-0.32(-0.70;0.06)	-0.41 (-0.79; -0.03)*	-0.23(-0.61;0.15)
	Slope	0.74(0.28;1.17)	1.03(0.46; 1.60)	1.32(0.71;1.90)
**J**	Intercept	0.13(-0.19;0.45)*	0.04 (-0.28; 0.35)	0.19(-0.13;0.51)*
	Slope	0.57(0.15;0.98)	0.55(0.09; 1.01)	0.44(-0.12;0.94)
**K**	Intercept	-0.27(-0.66;0.12)	-0.47 (-0.86; -0.08)*	-0.26(-0.65;0.13)
	Slope	1.28(0.74;1.83)	1.06(0.42; 1.71)	1.02(0.46;1.54)
**L**	Intercept	-0.81(-1.44;-0.17)*	-0.73 (-1.35; -0.10)*	-0.85(-1.48;-0.22)*
	Slope	1.13(0.48;1.79)	2.05(0.97; 3.23)	1.48(0.63;2.31)
**M**	Intercept	-0.06(-0.55;0.42)	-0.04 (-0.52; 0.44)	-0.13(-0.62;0.36)
	Slope	0.87(0.17;1.55)	0.83(-0.20; 1.83)	0.52(-0.46;1.28)
**N**	Intercept	0.20(-0.21;0.62)	0.10 (-0.32; 0.51)	0.29(-0.12;0.70)

	Slope	1.21(0.72;1.75)	1.33(0.81; 1.90)	1.15(0.54;1.74)
**O**	Intercept	0.54(0.05;1.03)*	0.80 (0.32; 1.28)*	0.72(0.23;1.20)*
	Slope	1.21(0.63;1.84)	1.99(1.12; 2.95)	1.99(1.23;2.84)
**P**	Intercept	-0.62(-2.02;0.78)	-0.65 (-2.05; 0.74)	-0.74(-2.14;0.66)
	Slope	-0.91(-4.02;1.73)	0.40(-7.58; 4.64)	-0.42(-6.90;3.45)

Calibration intercept and slope values were computed using the Cox approach (4).
* - denotes detected model miscalibration in either intercept or slope.

Supplementary Table 12. Predictor variables selected and their coefficients of final model fit using *central* and *FedAvg* model development strategy for 30-day TAVI post-operative mortality risk prediction models.

Model	Central	FedAvg
**Variable Name**	Coefficient	Coefficient
Intercept	-4.012	-16.079
Age	0.019	0.007
Body Mass Index (BMI)	-	0.009
Body Surface Area (BSA)	-1.358	-0.665
Log(Estimated glomerular filtration rate (eGFR))	-	0.272
Left Ventricular Ejection Fraction (LVEF)	-0.015	-0.005
Log(Pulmonary artery pressure (sPAP))	0.327	0.038
Log(Serum creatinine)	0.548	0.519
Access route direct aortic	-1.271	-0.596
Access route subclavian	-1.482	-0.905
Access route transapical	-1.075	-0.646
Access route transfemoral	-1.901	-1.074
Access route other	-	-
Anesthesia	0.312	0.235
Balloon pre-TAVI	0.109	-0.099
Canadian Cardiovascular Society (CCS) class IV angina	-0.416	-
Chronic lung disease	0.278	0.110
Diabetes Mellitus	-	0.006
Critical preoperative state	1.532	-
Dialysis	0.262	-
Extra-cardiac arteriopathy	0.070	0.053
Functional New York Heart Association (NYHA) class I	-	-
Functional New York Heart Association (NYHA) class II	-0.153	0.030
Functional New York Heart Association (NYHA) class III	0.215	0.106
Functional New York Heart Association (NYHA) class IV	0.538	0.280
Percutaneous Aortic Balloon Valvuloplasty (PABV)	-	-0.005
Poor mobility	-	0.034
Previous aortic valve surgery	0.248	-
Previous cardiac surgery	-	-0.056
Previous Cerebrovascular Accident (CVA)	-0.116	-0.072

Previous permanent pacemaker	-0.109	-
Procedure acuity elective	-	-
Procedure acuity emergency	-	0.270
Procedure acuity urgent	0.559	0.232
Recent Myocardial Infarction (MI)	0.275	-
Female Sex	-	0.034

Note: the log-transformed values were used in the case of serum creatinine, pulmonary artery pressure and estimated glomerular filtration rate. For display purposes, model parameter coefficients have been rounded to three digits after the decimal point.

Supplementary Table 13. Predictor variables selected and their coefficients of final model fit using *local* model development strategy for 30-day TAVI post-operative mortality risk prediction.

	Center													
**Variable**	**A**	**B**	**C**	**D**	**E**	**F**	**G**	**H**	**I**	**J**	**K**	**L**	**N**	**O**
	Coefficient													
Intercept	-2.03	-5.57	-6.45	-3.12	5.03	-10.33	0.25	-6.77	-5.39	-8.46	-5.92	9.25	-11.72	-3.59
Age	0.04	0.01	0.05	0.06	-0.04	0.04	0.04	-0.03						-0.03
BMI	-0.08	0.08	-0.09			-0.02	0.12	0.03	-1.74	0.04		-0.39		
BSA		-2.82		-1.07	-3.06	-1.11	-5.49						-3.57	-3.42
Log(eGFR)	-0.29			-0.45	-0.81			0.08				-0.95		
LVEF	-0.03	-0.03		-0.04	-0.02		-0.03		-0.02		-0.04			-0.03
Log(sPAP)		0.73	0.53	0.35	1.19	0.69				1.13				
Log(Serum creatinine)		0.69				0.79		0.84	1.41		0.94			2.35
Access route DA	-0.47												16.09	
Access route TA				0.85			-0.39							
Access route SC								-0.30						
Access route TF	-0.63			-0.58			-0.78	-0.92	-0.76	-0.61			14.82	
Access route other														
Anesthesia	0.93	0.92		0.26			1.31							
Balloon pre-TAVI		0.60	-0.91	-0.42			0.79			0.52				
Chronic lung disease	0.86	0.14	0.64							0.49				
Diabetes Mellitus		0.33	0.76											
ECA		-0.19	0.23	0.66	0.15	0.77								
NYHA class I														
NYHA class II							2.47							
NYHA class III						0.58	2.97	0.82	-0.38	0.01				
NYHA class IV										1.28				
Procedure acuity EL														
Procedure acuity EM														
Procedure acuity UR	0.55		0.36			1.05		1.57						
PABV		-0.44		0.31										
PCS			0.36	-0.44										
Previous CVA				0.68										
Poor mobility		0.14						1.01						
Female Sex		0.08					-0.98			0.28				

BMI – Body Mass Index (kg/m^2), BSA – Body Surface Area (m^2), eGFR – estimated Glomerular Filtration rate,
LVEF – Left-Ventricular Ejection-Fraction, *sPAP* – Systolic Pulmonary Artery Pressure,
Access route DA – Direct Aortic , *Access route TA*- Transapical,
Access route SC –Subclavian, *Access route TF* - Transfemoral, *ECA* - Extra-cardiac arteriopathy,
NYHA class - Functional New York Heart Association classification, *Procedure acuity EL* - Elective,
Procedure acuity EM- Emergency, *Procedure acuity UR* - Urgent, *PABV* - Percutaneous Aortic Balloon Valvuloplasty,
PCS – Previous cardiac surgery, *Previous CVA* – Previous Cerebrovascular Accident.
The columns for two centers (M and P) where no final model could be fit are not shown.
The log-transformed values were used in the case of serum creatinine, pulmonary artery pressure and estimated glomerular filtration rate. For display purposes, model parameter coefficients have been rounded to three digits after the decimal point.
Note: the selected variables and their coefficients also apply for the local models used by the final *ensemble* model development strategy.

Supplementary Table 14. Hyperparameter values selected of final model fits for 30-day TAVI post-operative mortality risk prediction models from four model development strategies (*central*, *local*, *FedAvg*, *ensemble*).

Model Strategy	Center	Hyperparameter	Value
**Central**	NA	LASSO lambda	0.0007
**FedAvg**	NA	Agreement Strength	0.00
	NA	Epochs	500
	NA	Learning rate	0.010
	Center A	LASSO lambda	0.003
	Center B	LASSO lambda	0.002
	Center C	LASSO lambda	0.004
	Center D	LASSO lambda	0.003
	Center E	LASSO lambda	0.003
	Center F	LASSO lambda	0.001
	Center G	LASSO lambda	0.003
	Center H	LASSO lambda	0.005
	Center I	LASSO lambda	0.009
	Center J	LASSO lambda	0.003
	Center K	LASSO lambda	0.015
	Center L	LASSO lambda	0.004
	Center O	LASSO lambda	0.008
	Center P	LASSO lambda	0.010
**Local**	Center A	LASSO lambda	0.003
	Center B	LASSO lambda	0.002
	Center C	LASSO lambda	0.004
	Center D	LASSO lambda	0.003
	Center E	LASSO lambda	0.003
	Center F	LASSO lambda	0.001
	Center G	LASSO lambda	0.003
	Center H	LASSO lambda	0.005
	Center I	LASSO lambda	0.009
	Center J	LASSO lambda	0.003
	Center K	LASSO lambda	0.015
	Center L	LASSO lambda	0.004
	Center O	LASSO lambda	0.008
	Center P	LASSO lambda	0.010
**Ensemble**	Center A	LASSO lambda	0.003

Center B	LASSO lambda	0.002
Center C	LASSO lambda	0.004
Center D	LASSO lambda	0.003
Center E	LASSO lambda	0.003
Center F	LASSO lambda	0.001
Center G	LASSO lambda	0.003
Center H	LASSO lambda	0.005
Center I	LASSO lambda	0.009
Center J	LASSO lambda	0.003
Center K	LASSO lambda	0.015
Center L	LASSO lambda	0.004
Center O	LASSO lambda	0.008
Center P	LASSO lambda	0.010

Note: For the LASSO lambda regularization parameter the considered values were automatically chosen by the cv.glmnet function from the glmnet library in R.
For the agreement strength parameter, considered values were 0, 0.25, 0.5, 0.75, and 1.
For the learning rate parameter, considered values were 0.01, 0.05, and 0.1.
For the number of training epochs parameter, considered values were 50, 200, 500, and 1000.
NA = Not Applicable.

Supplementary Table 15. Net Reclassification Improvement results from Cross-Validation for 30-day TAVI post-operative mortality risk prediction models from four model development strategies (*central*, *local*, *FedAvg*, *ensemble*).

**Model Strategy**	Central	Local	FedAvg	Ensemble
**Case-positive group**				
Central		0.06	0.01	0.01
Local	-0.06		-0.05	-0.04
FedAvg	-0.01	0.05		0.00
Ensemble	-0.01	0.04	0.00	
**Case-negative group**				
Central		-0.03	0.10	0.20
Local	0.03		0.10	0.14
FedAvg	-0.10	-0.10		0.09
Ensemble	-0.20	-0.14	-0.09	

Supplementary Table 16. Net Reclassification Improvement (NRI) results from Leave-Center-Out Analysis (LCOA) for 30-day TAVI post-operative mortality risk prediction models from four model development strategies (*central*, *local*, *FedAvg*, *ensemble*).

**Model Strategy**	Central	FedAvg	Ensemble
**Case-positive group**			
Central		0.04	0.02
FedAvg	-0.04		-0.02
Ensemble	-0.02	0.02	
**Case-negative group**			
Central		0.08	0.14
FedAvg	-0.08		0.07
Ensemble	-0.14	-0.07	

4 Appendix D: Supplementary Methods and Results

Supplementary Methods 1. Variable selection for *FedAvg*

As previously-described in the methods section, for each model development strategy, predictor variables were selected from the set of available candidate predictor variables via least absolute shrinkage and selection operator (LASSO).
In the case of the central model, given a multicenter training dataset DS_{train}, LASSO was applied on DS_{train}, and the variables selected from this ($VS_{central}$) were set as the predictors to use for fitting the model on DS_{train}.
In the case of local models, the multicenter dataset DS_{train} would first be partitioned into *h* single-center training datasets (DS_{train_1}, … , DS_{train_h} , where *h* is the number of hospitals in DS_{train}).
Thereafter, LASSO would be applied on each center-specific dataset and hence produce h sets of selected variables (VS_{local_1}, …, VS_{local_h}), one per center.
The same process for variable selection as for local was also used for ensemble.
For FedAvg , we first followed the same steps as just mentioned for local and ensemble to obtain a set of center-specific variable selections (VS_{FedAvg_1}, … , VS_{FedAvg_h}).
Next, the *h* sets of selected variables were combined into a single set $VS_{FedAvg\_Combined}$.
This was done via the agreement strength (*AgrStr*) hyperparameter in the following manner:
1) compute the volume weight (*vw*) of each participating hospital *H* in DS_{train} by its proportion of records in DS_{train} (e.g., if DS_{train} contains 1000 records, and hospital A has 500 records in DS_{train} , then the weight for hospital A is 0.5).

2) for each candidate variable ($cvar_1$, …, $cvar_i$, where i is the number of candidate predictors) in DS_{train} , calculate the volume-weighted frequency of occurrences it was selected in all center-local variable sets ($cvar_1$ in $VS_{FedAvg\_1}*vw_1$, … , $cvar_1$ in $VS_{FedAvg\_h} *vw_h$, … , $cvar_i$ in $VS_{FedAvg\_1}*vw_1$, … , $cvar_i$ in $VS_{FedAvg\_h} *vw_h$).

3) Derive the combined set of selected variables $VS_{FedAvg\_Combined}$ as the candidate predictors whose volume-weighted frequency of occurrence is greater than or equal to *AgrStr*.

Note: We can think of the AgrStr hyperparamter as defining a spectrum between the set union and the set intersection operations, where a AgrStr of zero would indicate a union (i.e., select variables that we selected by at least one hospital-local LASSO), and a AgrStr of one would denote a intersection (i.e., select only variables that were present in all hospital-local LASSOs).

To illustrate this with an example, suppose the variable for chronic lung disease (CLD) was selected from LASSO in two hospitals (A and B), and the variable dialysis was selected in 2 hospitals (B and C). In this example, let DS_{train_A} have 500 records, DS_{train_B} have 300 records, and DS_{train_C} have 200 records, and thus DS_{train} contains a total of 1000 records. Let the AgrStr hyperparameter have been set to 0.75 in this example. From this we can calculate that volume weight of hospital A is, vw_A = 500/1000 = 0.5, and for hospital B vw_B = 300/1000 = 0.3 , and for hospital C vw_C = 0.2 . For the variable CLD, we calculate it's weighted frequency will then be $vw_A + vw_B = 0.8$, while for the dialysis variable the weighted frequency will be $vw_B + vw_C = 0.5$. From this, we apply the threshold AgrStr of 0.75, and we derive the $VS_{FedAvg\_Combined}$ to contain only the variable CLD, and not dialysis.

Supplementary Methods 2. Net Reclassification Improvement measures.

The Net Reclassification Improvement (NRI) was calculated between the predictions of any two models in either validation strategy (CV and LCOA). For a given reference model A, the NRI quantifies the ability of model B to increase the probabilities of the event for patients with the event, and decrease these probabilities for those without the event. When comparing two models' predictions their NRI value will range from -1 to 1, with -1 indicating that for every record the prediction of the first model is worse (further from the actual outcome) than that of the second model, and 1 indicating that every prediction of the first model is better (closer to the outcome) than that of the second model. Prior to calculating the NRI, model predictions were rounded to two digits after the decimal point to avoid superfluous comparisons (i.e., we considered a model predicted risk of, e.g., 0.034 to be no different from a predicted risk of 0.031).We computed NRI values per model pair per outcome and presented the results in four NRI matrices.

Supplementary Methods 3. Hyperparameter optimization

Hyperparameter optimization was performed using grid search on the training set, which itself was divided into five folds. Each fold consisted of an 80% training subset and a 20% evaluation subset. For each combination of hyperparameter values, a model was trained on the 80% subset and its performance, measured by AUC, was evaluated on the 20% subset. The mean AUC across all five evaluation subsets was calculated for each set of hyperparameter values. The optimal hyperparameter set was chosen based on the highest mean AUC. Ties were resolved by selecting the hyperparameter set with the lowest variation in AUC.

Supplementary Methods 4. Imputation of missing values.

Given a dataset DS with some missing values, we imputed one copy of DS using Chain Equations (5). For the central model development strategy we imputed directly on the whole DS (Note: during any model validation strategy, before any imputation was done we would first split the dataset into train and test sets, and impute on each separately).
For the other model strategies (local, FedAvg, ensemble), we first partitioned the dataset into *h* single-center datasets (DS_1, … , DS_h , where *h* is the number of hospitals in DS). Thereafter, we proceeded to impute one copy of each center-specific dataset separately. In this way, we preserved constraint for data to not be shared between hospitals in the federated and local model strategies. Note: One could also learn a common imputation model between different locations using a federated learning approach (6), however this was not investigated in the current research.

Supplementary Results and Discussion

Supplementary Results 1. Net Reclassification Improvement (NRI) results.

Results Comparing model predictions using the net reclassification improvement (NRI) during Cross-Validation (CV) showed that the predictions from the *central* model in the case-positive

outcome group offered a reclassification improvement over the predictions of all other models. The *ensemble* and *FedAvg* predictions had no difference between each other (NRI was zero). Both *FedAvg* and *ensemble* predictions offered a reclassification improvement over those from *local* (Supplementary Table 15).

For the case-negative outcome group (i.e., when the patient was still alive 30 days after their TAVI procedure), *local* predictions had a reclassification improvement over all other models. *Central* had an improvement over the two federated approaches, and *FedAvg* offered an improvement over *ensemble.*

NRI measures from the Leave-Center-Out analysis (LCOA) confirmed almost the same arrangement as we saw from CV with the expected exclusion of *local* models. In addition, for the positive group, *ensemble* now showed a reclassification improvement over *FedAvg* (Supplementary Table 16).

For the case-negative group of the LCOA, *central* was shown as superior to both federated approaches, while *FedAvg* was shown as superior to *ensemble*.

Discussion For the outcome-negative group during CV *local* model predictions were seen as the best, followed by central, *ensemble*, and *FedAvg*.

The higher comparative NRI of *local* models for case-negative records simply shows that for the same patient with a negative outcome *local* models predicted on average a lower risk probability than the other models. In an extreme example, if a new model were to predict only zero risk probabilities, it would have a superior NRI to all other models in the case-negative group (and also an inferior NRI to all others in the case-positive group). While *local* models showed superior NRI to *central* and federated ones in the case-negative group, it should be noted that *local* models also performed with poorer discrimination as measured by the AUC metric. This means that if one were to examine the predicted risks of the *local* model for two hypothetical patients, one with a positive outcome and one with a negative outcome, the *local* model would make the mistake of assigning a higher risk probability to the case-negative patient more often than either federated or central models.

Supplementary Results 2. Sensitivity Analyses results.

Federated learning without recalibration Training the *FedAvg* and *ensemble* models without recalibration had no impact on their AUC performance, however both models performed notably worse in calibration performance in both internal and external validations.

The calibration intercept of both model types was further away from zero (the ideal intercept value) when compared to their corresponding recalibrated model versions (-0.23 [95%CI -0.32;-0.13] versus -0.04 [95%CI -0.07; -0.02] for *FedAvg* during CV) (-0.28 [95%CI -0.38;-0.19] versus -0.04 [95%CI -0.06; -0.01] for *ensemble* during CV). The calibration graphs showed both FL model predictions to now deviate further from perfect calibrations with signs of model over-prediction.

Without recalibration applied, both FL strategies outperformed the central model in terms of NRI in the outcome-positive record group with *FedAvg* also outperforming *ensemble*. This was the case for both CV and LCOA. In terms of the outcome-negative record group *central* maintained its top position, but *FedAvg* now outperformed *local* in CV. Also in the case-negative group for CV *ensemble* without recalibration performed the worst. In the LCOA, *central* remained first (in both negative and positive outcome groups), while NRI for *FedAvg* improved and NRI for *ensemble* worsened.

Excluding low-volume hospitals In the second sensitivity analysis three hospitals were removed from the dataset (P, O, N) for not having a sufficient volume of TAVI records as defined in Section 2.4.6.

In this analysis mean AUC of *central* dropped by 0.01 during CV and LCOA.
Similarly, the mean AUC of *FedAvg* dropped by 0.02 during both validations.
For *ensemble*, the mean AUC did not change during CV and dropped by 0.01 during LCOA.
Hospital-*local* models showed a mean AUC that was 0.04 lower than from the main analysis.
From numerically testing model predictions for miscalibration via the Cox approach (4) during CV *FedAvg* had no detected miscalibration, *central* and *ensemble* were miscalibrated by slope, and *local* was miscalibrated by intercept, compared to the main results wherein all models showed miscalibration.
In the LCOA, both federated models were not miscalibrated and the *central* model was miscalibrated by slope, compared to the main results wherein no models were miscalibrated.

Supplementary References

1. McMahan B, Moore E, Ramage D, Hampson S, Arcas BAy. Communication-Efficient Learning of Deep Networks from Decentralized Data. In: Aarti S, Jerry Z, editors. Proceedings of the 20th International Conference on Artificial Intelligence and Statistics; Proceedings of Machine Learning Research: PMLR; 2017. p. 1273--82.
2. (NHANES) NHaNES. Centers for Disease Control and Prevention (CDC); 2018.
3. Efron B. Bootstrap Methods: Another Look at the Jackknife. The Annals of Statistics. 1979;7(1):1-26.
4. COX DR. Two further applications of a model for binary regression. Biometrika. 1958;45(3-4):562-5.
5. van Buuren S, Groothuis-Oudshoorn K. mice: Multivariate Imputation by Chained Equations in R. Journal of Statistical Software. 2011;45(3):1 - 67.
6. Balelli I, Sportisse A, Cremonesi F, Mattei P-A, Lorenzi M. Fed-MIWAE: Federated Imputation of Incomplete Data via Deep Generative Models. arXiv [statML]. 2023;abs/2304.08054.
